# Development and validation of an algorithm to predict the treatment modality of burn wounds using thermographic scans: Prospective cohort study

**DOI:** 10.1371/journal.pone.0206477

**Published:** 2018-11-14

**Authors:** Mario Aurelio Martínez-Jiménez, Jose Luis Ramirez-GarciaLuna, Eleazar Samuel Kolosovas-Machuca, Justin Drager, Francisco Javier González

**Affiliations:** 1 Department of Surgery, Faculty of Medicine, Universidad Autónoma de San Luis Potosí, San Luis Potosí, SLP, Mexico; 2 Burn Unit, Hospital Central Dr. Ignacio Morones Prieto, San Luis Potosí, SLP, Mexico; 3 Doctorado Institucional en Ingeniería y Ciencia de Materiales (DICIM-UASLP), Universidad Autónoma de San Luis Potosí, San Luis Potosí, SLP, Mexico; 4 Division of Experimental Surgery, Faculty of Medicine, McGill University. Montreal, QC, Canada; 5 Coordinación para la Innovación y Aplicación de la Ciencia y la Tecnología, Universidad Autónoma San Luis Potosí, San Luis Potosí, SLP, Mexico; US Army Institute of Surgical Research, UNITED STATES

## Abstract

**Background:**

The clinical evaluation of a burn wound alone may not be adequate to predict the severity of the injury nor to guide clinical decision making. Infrared thermography provides information about soft tissue viability and has previously been used to assess burn depth. The objective of this study was to determine if temperature differences in burns assessed by infrared thermography could be used predict the treatment modality of either healing by re-epithelization, requiring skin grafts, or requiring amputations, and to validate the clinical predication algorithm in an independent cohort.

**Methods and findings:**

Temperature difference (ΔT) between injured and healthy skin were recorded within the first three days after injury in previously healthy burn patients. After discharge, the treatment modality was categorized as re-epithelization, skin graft or amputation. Potential confounding factors were assessed through multiple linear regression models, and a prediction algorithm based on the ΔT was developed using a predictive model using a recursive partitioning Random Forest machine learning algorithm. Finally, the prediction accuracy of the algorithm was compared in the development cohort and an independent validation cohort. Significant differences were found in the ΔT between treatment modality groups. The developed algorithm correctly predicts into which treatment category the patient will fall with 85.35% accuracy. Agreement between predicted and actual treatment for both cohorts was weighted kappa 90%.

**Conclusion:**

Infrared thermograms obtained at first contact with a wounded patient can be used to accurately predict the definitive treatment modality for burn patients. This method can be used to rationalize treatment and streamline early wound closure.

## 1. Introduction

Burn injuries are the fourth most common type of trauma worldwide with an estimated 300,000 deaths occurring annually secondary to these injuries. For survivors, burns can result in significant morbidity and permanent disability with a major impact on their quality of life [1]. To mitigate these consequences, thorough initial wound assessment is critical to predict healing outcomes and to guide the optimal clinical decision-making. However, even for the most experienced clinicians, the subjective assessment of the visual and tactile characteristics of the tissue predicts the severity of the injury in only 50 to 70% of cases [2–4]. Moreover, accuracy also depends on the availability of trained personnel, their clinical experience, and on burn conversion, a phenomenon by which some superficial partial-thickness burns spontaneously become deep partial-thickness or even full-thickness wounds [2, 5–7]. Over time, burn conversion increases the extent of tissue damage in a way that may not be immediately evident, thus confounding the initial assessment of the wound. A more objective measurement of burn severity could give clinicians a secondary tool to more accurately and rapidly assess burn severity and streamline definitive treatment to accelerate patient recovery and rehabilitation.

Digital infrared thermography imaging can be used to assess the severity of burns in a non-invasive manner. This imaging modality may provide more information about the degree of tissue damage than clinical inspection alone during the various phases of wound healing [8] and can quantitatively assess the burn depth based on the digital images acquired [9]. Previous studies have shown that the destruction of blood vessels at the time of skin injury leads to a reduction in local perfusion, and a secondary lowering of the skin temperature at that area [10–12]. This amount of disruption of blood flow correlates with the extent of the injury and is displayed as a colour map of the wound after imaging with infrared thermography. The colour maps are then used to measure the skin temperature at various areas and allow the calculation of a delta-T (ΔT), representing the difference in temperature between the wound site and an adjacent healthy skin region [10]. This value, which represents the temperature difference between the two body areas acquired by static imaging, has been correlated with tissue viability, the healing potential of the wound, and has been found to possess excellent inter-observer reliability [6, 13]. As such, thermography has the potential to be used as a bedside diagnostic tool to predict the treatment modality needed to achieve early wound closure. However, this strategy has yet to be validated.

In this study, we developed a model to predict the treatment modality needed to promote closure of extensive skin burns in limbs based on thermographic imaging of the wound obtained during the first three days of treatment. We hypothesized that the temperature difference between the healthy and wounded skin correlates with the required definitive treatment in a cohort of burn patients. We categorized the treatment as “re-epithelization” if the wound healed spontaneously, “skin graft” if the injury healed after receiving a graft, or “amputation” if the patient required removal of part of an appendage because of lack of tissue viability. Subsequently, by using the ΔT values, we created a prediction model based on temperature difference cut offs for each treatment modality. Finally, we tested and validated the prediction model in an independent cohort of similarly burned patients.

## 2. Patients and methods

### 2.1. Study design

This was a prospective observational study. It was approved by the Ethics Committee of Hospital Central “Dr. Ignacio Morones Prieto” in San Luis Potosi, Mexico (registry 16–17). All clinical investigations were conducted according to the principles expressed in the Declaration of Helsinki. All patients agreed to participate and provided informed consent. In the case of children, consent was obtained from their parent or guardian, and they provided their assent to participate.

### 2.2. Patients

All study patients received treatment at the burn care unit of Hospital Central “Dr. Ignacio Morones Prieto,” a major referral centre for burn injuries in central Mexico. Patients were consecutively selected providing they met all inclusion criteria and had none of the exclusion criteria. Inclusion criteria were patients having sustained partial or full thickness burns in extremities covering >25 cm^2^ of the total body surface and who were admitted to the burn care unit within 24 hours from injury. Exclusion criteria were the presence of any previous comorbidity, a baseline body mass index of <19.9 for adults or below the 5^th^ percentile for their age in children, the presence of foreign bodies embedded in the tissue, gross oedema, systemic causes of distal hypoperfusion, or presence of local infection. No patients were eliminated from the study after enrolment. Two independent prospective cohorts were used for this study: a cohort used to develop the prediction algorithm and a cohort used to test its performance. Regardless of the cohort, all imaging and initial treatment were done identically to all patients as described below.

### 2.3. Infrared imaging

Infrared thermography was performed once within the first three days after injury, as this timeframe is the most informative for assessing wound characteristics [14]. Imaging was done at the bedside, as the burn unit is considered a sterile hospital area.

Before imaging, the dressing was removed, and the wound was cleaned with a 5% chlorhexidine solution, rinsed with 0.9% saline, and dried with sterile gauze. Loose skin was then removed, along with any blisters present and the wound was allowed to reach room temperature for 3 minutes. All the temperature measurements were taken following the Thermographic Imaging in Sports and Exercise Medicine (TISEM) check list [15], at a distance of 0.5 or 1.5 m, whichever distance was best to capture the whole extent of the burned tissue, at an angle of 90° relative to the body, in a closed room under controlled conditions of light and external radiation exposure, at controlled room temperature (22°C) and atmosphere humidity of 40%. We followed the Glamorgan protocol [16], which defines the regions of interest that must be measured by thermography in a human body to guarantee repeatability. Briefly, this protocol consists in an atlas of skin temperature distribution in 90 body regions of interest that is used as a pattern to reproduce the views of body positions to increase the repeatability of thermal imaging.

Static digital infrared thermographic images were acquired with a FLIR T400 infrared camera (FLIR System, Wilsonville, OR, 2013) with a 320 x 240 focal plane array of uncooled microbolometers, a spectral range of 7.5 to 13 μm, and a thermal sensitivity of 50 mK at 30°C. The camera was left on for 5 minutes before acquiring the images to allow stabilization of the sensor. The skin emissivity was set at 0.98 for all the acquired measurements. After imaging, the wounds were re-dressed and received standard care.

Thermographic analysis of the images was performed using the FLIR Tools Quick-Report v.1.2 software (FLIR Systems, version 5.70, 2016), which includes a tool to obtain the maximum, minimum, and average temperature of a user-defined area. An investigator blinded to the clinical characteristics of the wound delineated a region of interest (ROI) corresponding to the burn area using a phantom of the clinical image and the thermogram, and the software was then used to obtain the maximum, minimum, and average temperature of the ROI. A 25 cm^2^ region was delineated on the healthy skin adjacent to the wound and the same measurements performed. Both mean temperatures were recorded, as well as the difference between them, which is the ΔT (S1 Fig).

### 2.4. Wound treatment modality

All wounds were independently stratified by two experienced surgeons immediately after admission into one of 3 categories: superficial partial thickness, deep partial thickness, and full thickness, which also included burns to underlying tissue (fourth degree burns) [17]. No discrepancies were found between their assessments. All burns received the standard treatment according to the International Society for Burn Injuries (ISBI) guidelines by a surgical team blinded to the thermograms and prediction data: cleansing the wound every 72 hours, early excision of necrotic tissue, wound coverage with silver sulfadiazine, and no antibiotic prophylaxis [18].

All wounds were followed for 15 days, after which the decision to graft or not was made based on the clinical characteristics of the wound. In the case of amputations, the decision to amputate and the procedures were done within 5 to 7 days of admission of the patient into the burn unit, based on the ISBI guidelines [18].

The modality of wound treatment was defined as “re-epithelization” if the wound re-epithelized by itself before 15 days of care; “skin graft” if the wound healed after receiving one or more skin grafts (all patients received auto-grafts), or “amputation” if the appendage was removed. We recorded the final wound modality, for example, if a wound received a graft but the extremity became nonviable and was amputated, it was considered as an amputation.

### 2.5. Statistical analysis

Data are expressed as the mean and standard deviation or proportions, as appropriate. Statistical analysis was performed using the statistical package R v.3.3.2 (R Core Team, Vienna, Austria, 2016) and RStudio (RStudio Team, Boston, MA, 2016). A power analysis was performed based on results of a previous study [11]. We determined that a minimum of 10 patients per outcome group was needed to detect a difference of 2.0 ± 1.5°C between treatment groups at an alpha level of 0.05 and a statistical power of 80%. Analysis of variance (ANOVA) and linear regression was used to compare temperature difference ΔT and identify potentially confounding factors (age, sex, burn aetiology, site of injury, depth of injury, burned area, and time of ΔT measurement). Multiple linear regression models were used to adjust for the significant confounding factors identified in the bivariate regression analysis. In all required cases, Tukey *post-hoc* tests were used to perform multiple comparisons. For the development of the prediction model, receiver-operator characteristic (ROC) curves were used, as well as machine learning predictive modelling by recursive partitioning Random Forest algorithms and unsupervised k-means clustering. Finally, to test the agreement rate between the prediction model and the treatment modality we used weighted kappa analysis.

## 3. Results

### 3.1. Patient characteristics

**Fig 1:**
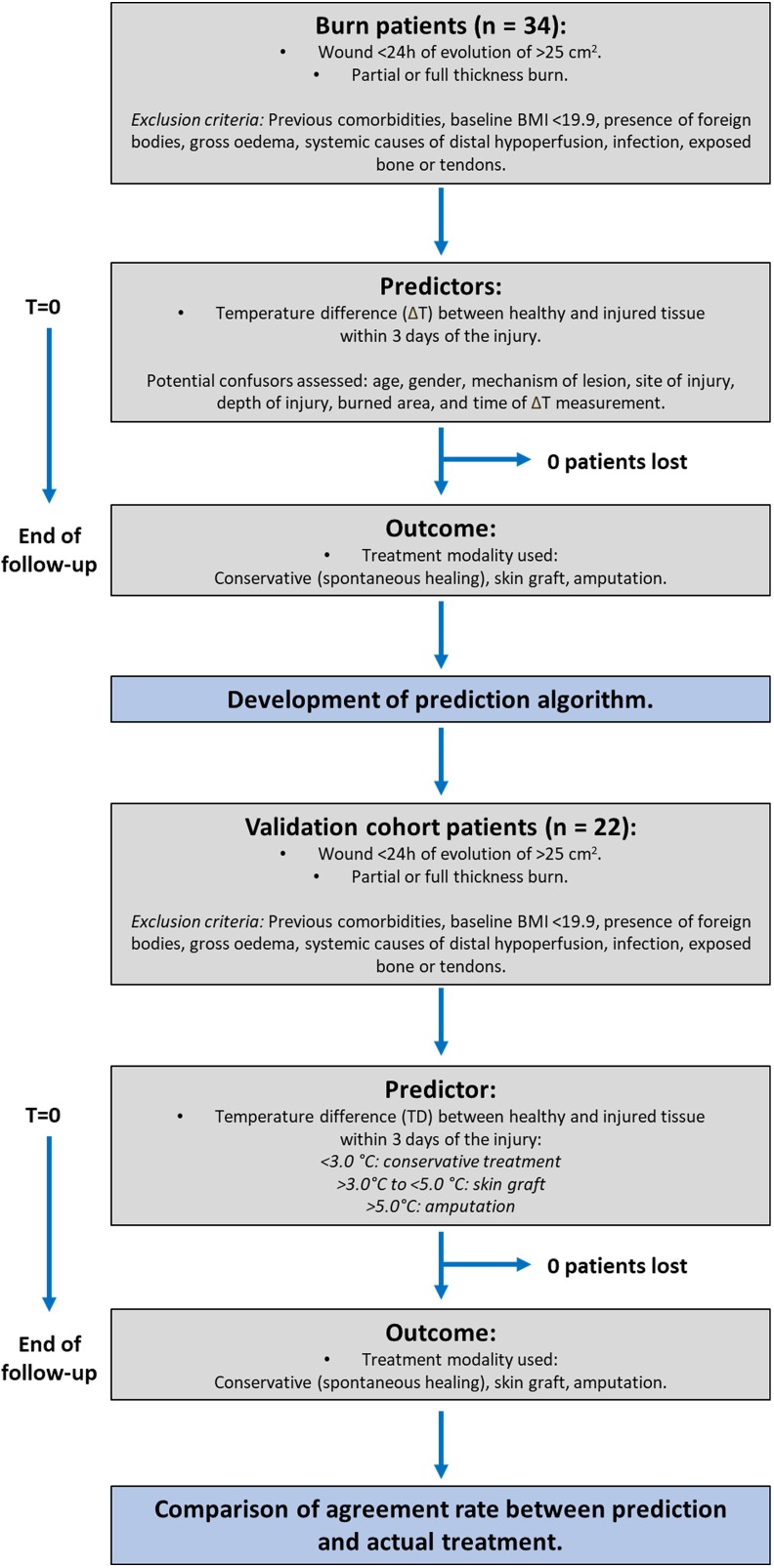
Study flowchart.

The flowchart summary of the study is shown in Fig 1 and the patient characteristics in Table 1. Two independent patient cohorts were used in this study, one to train the predictive model (development cohort, n = 34), and a second one to validate the findings (validation cohort, n = 22).

**Table 1:** Patient characteristics.

Variable		Development cohort	Validation cohort	p value
**Age**	Mean (SD)	24 (17.9) years	26.5 (19.4) years	**0.746**
**Gender**	Women	7 (20%)	6 (27%)	**0.563**
	Men	27 (80%)	16 (73%)	
**Wound etiology**	Scald	12 (35%)	6 (27%)	**0.615**
	Fire	15 (45%)	9 (41%)	
	Electricity	7 (20%)	7 (32%)	
**Site of injury**	Upper limb	19 (56%)	17 (77%)	**0.103**
	Lower limb	15 (44%)	5 (33%)	
**Depth of injury**	Superficial partial thickness	10 (30%)	6 (27%)	**0.584**
	Complete partial thickness	3 (9%)	4 (19%)	
	Full thickness	21 (61%)	12 (54%)	
**Time of ΔT measurement**	Mean (SD)	1.45 (0.8) days after injury	1.86 (0.7) days after injury	**0.061**
**Burn area**	Mean (SD)	178.2 (198.4) cm^2^	392.3 (753.2) cm^2^	**0.560**
**Wound temperature (°C)**	Mean (SD)	29.6 (4.4)	29.3 (6.1)	**0.869**
**Uninjured skin temperature (°C)**	Mean (SD)	33.8 (2.1)	33.1 (4.6)	**0.941**
**Treatment modality**	Conservative	13 (37%)	9 (41%)	**0.977**
	Skin graft	10 (30%)	6 (27%)	
	**Amputation**	**11 (33%)**	**7 (32%)**	

Values are presented as median and standard deviation or proportions. P values represent comparisons between values for the development cohort vs. validation cohort. Tests were done with ANOVA for continuous data or Fisher exact tests for categorical data.

### 3.2. Determining if ΔT correlates with the definitive treatment of burn

A total of 34 patients (mean age 26.5 ± 19.4 years, min 1, max 68) were used to develop the prediction model (development cohort). From them, 14 (41%) were children. From the full cohort, 13 (39%) had partial-thickness burns, and 21 (61%) had full-thickness burns. Fifteen (45%) burns were caused by fire, 12 (35%) were scalds, and 7 (20%) were caused by electricity.

Thermographic measurements were obtained within the first three days of treatment (mean time 1.45 ± 0.8 days, median 1.0 day) and the ΔT calculated. ΔT in superficial partial thickness degree burns was 1.77 ± 0.92°C, 2.76 ± 1.05°C in deep partial thickness degree burns, and 5.45 ± 2.86°C in full thickness degree burns (p = 0.791 in superficial partial vs. deep partial thickness, p <0.001 in superficial partial vs. full thickness, and p = 0.170 in deep partial vs. full thickness).

**Fig 2:**
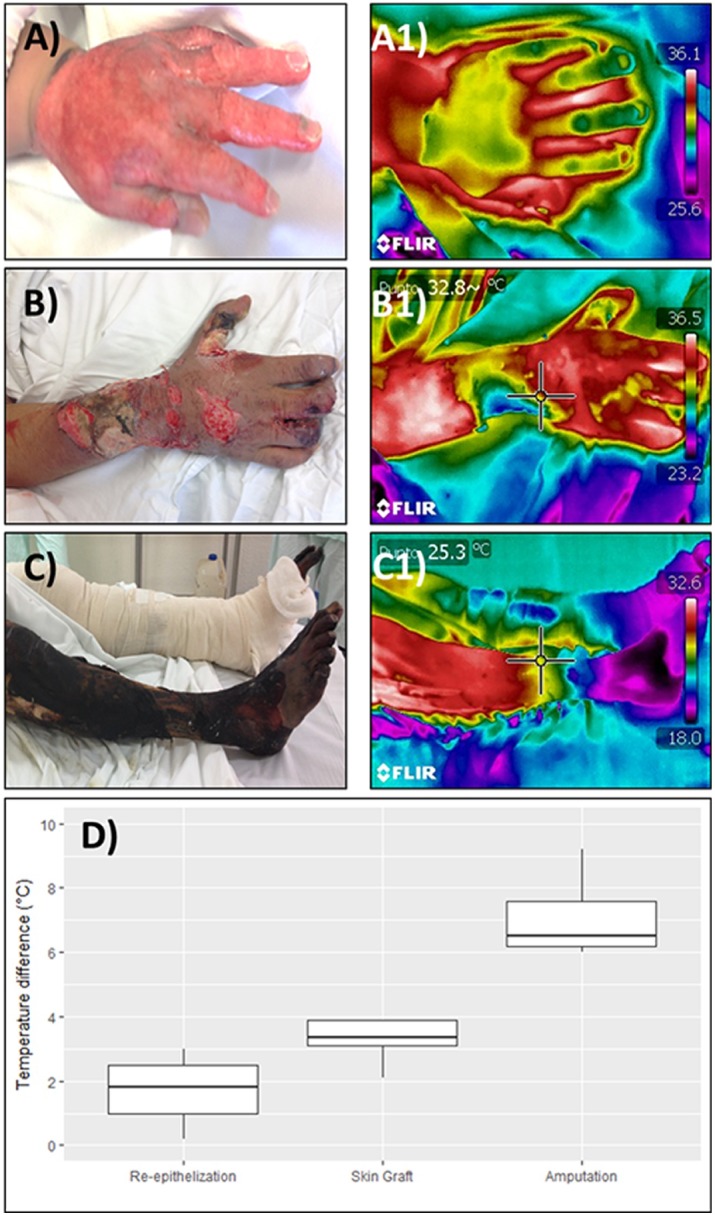
Thermograms and treatment groups. Clinical images (A to C) and thermograms (A1 to C1) were obtained during the first three days after the injury, the patients were followed up until discharged, and their outcome classified as healed by conservative treatment, skin graft or amputation. Significant differences in the temperature difference (TD) between injured and healthy tissue across all groups were found (D). Panels A and A1 represent an injury that healed by epithelization, B and B1 one that required a skin graft (note the colder region on the ulnar surface of the forearm), and C and C1 an injury that required amputation. Noteworthy in this last image, even though all the leg skin looks charred in the clinical image, the thermogram suggested that only the feet were non-viable.

Patients were followed-up until discharge and their outcome registered. Thirteen patients (37%) healed by re-epithelization, 10 (30%) received skin grafts, and 11 (33%) required an amputation. ΔT in patients who received conservative treatment was 1.75 ± 0.89°C, 3.28 ± 0.68°C in patients who received skin grafts, and 7.71 ± 1.89°C in patients who underwent amputation. Significant differences were detected among all groups (p <0.01 in all cases, Fig 2).

To characterize potential confounding factors that could be associated to ΔT (dependent variable), bi-variate linear regression models were performed for the following independent variables: age, sex, burn aetiology, site of injury, depth of injury, burned area, and time of ΔT measurement. Subsequently, the variables found to be significantly associated with ΔT were input into a multiple linear model analysis. This statistical approach was decided due to the sample size of the development cohort to avoid overfitting the multiple linear model (Table 2).

**Table 2:** Confounding factors for ΔT.

Factor	Bi-variate p value	Multi-variate p value
Treatment modality	<0.001	<0.001
Age	0.02	0.62
Gender	0.18	
Burn etiology	<0.001	0.65
Site of injury	0.23	
Depth of injury	<0.001	0.16
Burn area	<0.001	0.97
Time of ΔT measurement	0.21	

Potential confounding factors associated with ΔT measurements were assessed through bi-variate linear analysis. Cofounders significantly associated with ΔT were then used in a multiple linear regression analysis. In the multi-variate analysis, only treatment modality remained significantly associated with ΔT.

Factors significantly associated with ΔT were age (0.06°C increase per 1 year increase, p = 0.026), burn aetiology (ΔT in scalds burns 1.84 ± 0.86, 6.22 ± 2.76 in fire burns, and 3.59 ± 2.06 in electrical burns; scalds vs. fire burns p<0.001, NS for other comparisons), depth of injury (ΔT in superficial partial thickness burns 1.77 ± 0.92 degrees, 2.76 ± 1.05 in deep partial thickness burns, and 5.45 ± 2.86 in full thickness burns; superficial second degree vs. deep second degree burns p = 0.791, p <0.001 for superficial second degree vs. third degree burns, and p = 0.170 for deep second degree vs. third degree burns), and burn area (0.01°C increase per 1 cm^2^ increase, p <0.001). Afterwards, all variables found to be significantly associated with ΔT were input in to the multiple linear regression analysis *ΔT ~ treatment modality + age + burn aetiology + depth of injury + burn area*. A series of likelihood tests were performed, dropping the variable least likely to be significant until all variables were significant. The final model was ΔT *~ treatment* (R^2^ = 0.807, p <0.001). Thus, we concluded that only the definitive treatment was significantly associated with ΔT after multiple variable adjustment.

### 3.3. Development of a prediction algorithm using ΔT

The optimal ΔT cut-off values for prediction of treatment based on binary outcomes were calculated using ROC curves. Cut-off values and their associated sensibility, specificity, predictive values and area under the curve are shown in Table 3.

**Table 3:** Sensitivity and specificity analysis.

Treatment modality	Cut-off value	Sensitivity	Specificity	Positive predictive value	Negative predictive value	Area under the curve
**Conservative vs. Graft**	3.0°C	80%	100%	100%	86.6%	92.3
**Conservative vs. Amputation**	4.5°C	100%	100%	100%	100%	1.0
**Graft vs. Amputation**	4.9°C	100%	100%	100%	100%	1.0

**Fig 3:**
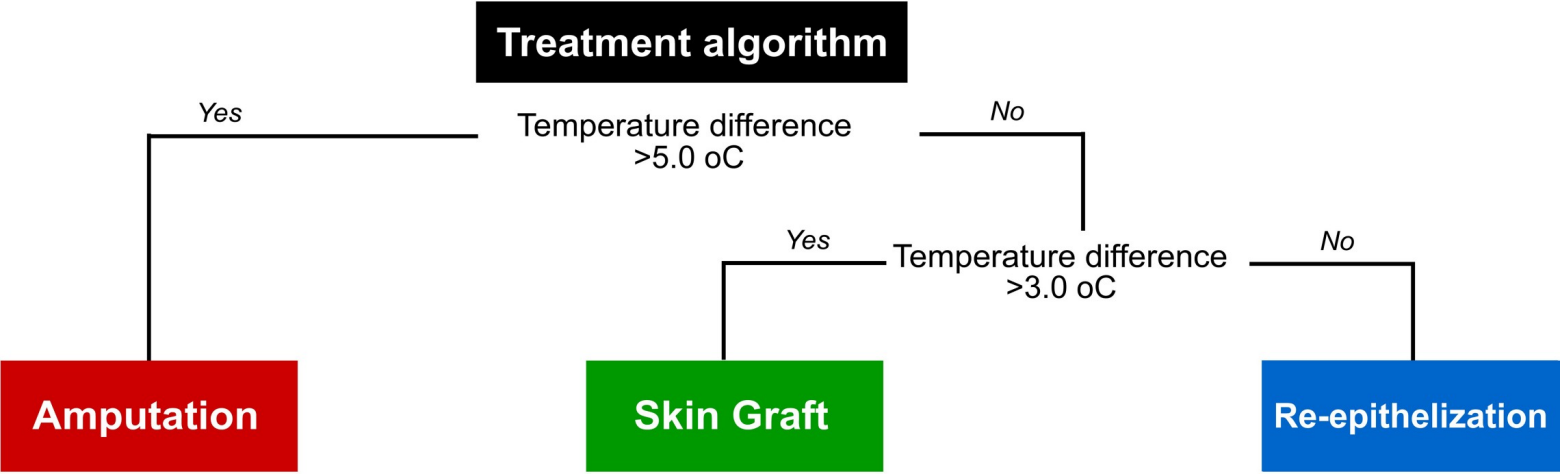
Clinical decision algorithm based on thermograms. Through Random Forest algorithms, the following clinical decision rule was developed: if a patient presents a temperature difference (ΔT) of >5.0°C, he will require amputation of the affected limb. If the ΔT is <5.0°C but >3.0°C, he will require a skin graft; and if the ΔT is <3.0°C, the wound will most likely heal by re-epithelization. This algorithm allows classification of the patient within the time of the first contact and has a theoretical accuracy of 85.35%.

Finally, to create the full decision-making model that included all 3 possible outcomes (re-epithelization, skin grafting or amputation), we used the Classification and regression training (caret) package for R [19] to create and validate a predictive model using recursive partitioning Random Forest algorithms to assign a treatment to patients based on their ΔT. The model had three classes (re-epithelization, skin graft or amputation) and five predictors (ΔT, age, burn aetiology, depth of injury, and burn area). The final model, which has the lowest complexity parameter value (S2 Fig), is presented in Fig 3. We tested the diagnostic accuracy of the model with 100 bootstrap resamples and found an accuracy of 85.35% (95%CI 72.2 to 98.5%) for diagnostic classification. The algorithm misclassified 13.0% of the patients to conservative treatment, 13.0% to skin graft, and 0% to amputation.

**Fig 4:**
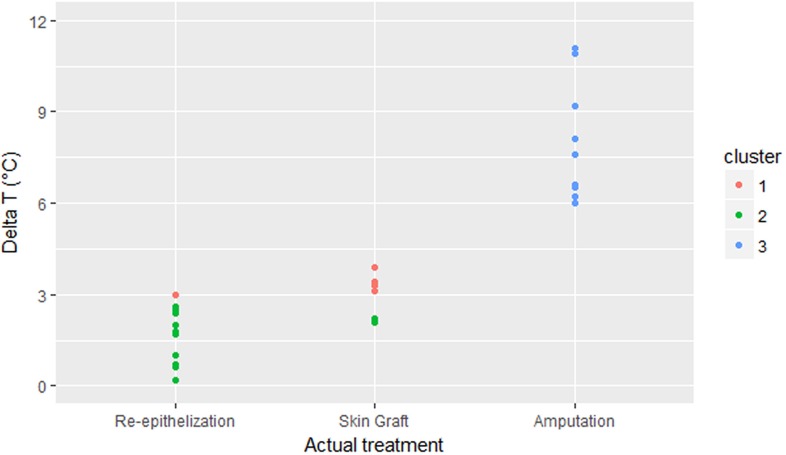
Unsupervised clustering of datapoints. Unsupervised k-means clustering of ΔT values of the development cohort was used to confirm the results of the Random Forest algorithm. In the x-axis of the graph, the three actual treatments used can be seen. Three clusters of datapoints arise based on the grouping of similar ΔT values. On the re-epithelization group, only one datapoint (green) lies outside of its cluster, while in the skin graft group, two datapoints (red) lie outside of their cluster. In the amputation group, all datapoints have been clustered together (blue). This technique supports the notion that a ΔT value of 3 and 5 correctly discriminates between treatment groups, regardless of the surgeon’s decision.

To control the fact that Random Forest algorithms is a type of supervised machine learning technique, which means that the method was trained to categorize the patients similarly as what the surgical team would have done, and to confirm the initial results of the algorithm, we conducted a second analysis using unsupervised k-means clustering of the ΔT values. Through this technique, the datapoints are grouped independently of the surgeon’s decision in such a way that the objects in the same cluster are more similar to each other than to the objects in other clusters. Results of the clustering, which support the cut-off values of the algorithm, are shown in Fig 4.

### 3.4. Agreement rate between predicted outcome and treatment modality

To test the prediction accuracy of the model in the development cohort, we obtained the inter-rater agreement weighted kappa coefficient. The algorithm predicted that 14 patients would heal by re-epithelization, 9 through skin grafts, and 11 would require an amputation. The treatment modality used on the patients was conservative treatment and re-epithelization in 13, skin graft in 10, and amputation in 11. Thus, the algorithm misclassified three patients (one in the re-epithelization group and two on the skin graft group). Agreement rate between the prediction and the patient outcome was weighted kappa = 0.904 (p <0.001).

### 3.5. Validation of the prediction algorithm

We prospectively enrolled 22 new patients with similar characteristics to develop a validation cohort. Clinical characteristics and their comparison to the development cohort are shown in Table 1.

After admission to the burn care unit and enrolment into the study, thermograms were acquired and analysed by an independent member of the research team. The surgical team was kept blinded to the prediction results until discharge of the patients when their treatment modality was recorded. The algorithm predicted that 9 patients would heal by re-epithelization with conservative treatment, 6 through skin grafts, and 7 would require an amputation. The true outcome of the patients was re-epithelization in 9 cases (1 wrongfully predicted to heal by grafting), skin graft in 6 (1 wrongfully predicted to heal by re-epithelization), and amputation in 7; thus, the algorithm misclassified two patients (Table 4). Agreement rate between the prediction and the patient outcome was weighted kappa = 0.901 (p <0.001).

**Table 4:** Agreement rate in the validation cohort.

	Predicted treatment			
**Treatment used**		**Re-epitdhelization**	**Skin graft**	**Amputation**
	**Re-epithelization**	8	1	0
	**Skin graft**	1	5	0
	**Amputation**	0	0	7

Agreement rate between the prediction algorithm and the treatment modality used in the patients of the validation cohort (n = 22) was found to be 90% (p <0.001), identical to the observed in the development cohort.

## 4. Discussion

Wound healing is a dynamic and complex biological process where it is commonly accepted that the original depth of the wound is not static. This is especially true for burns, where a variety of pathophysiologic mechanisms may cause a wound to progress into deeper tissue damage over the first few days after the injury [20]. One of the most common causes for wound conversion is the lack of an adequate blood supply, which leads to ischemia and autophagy of the surrounding tissue [20, 21]. As these phenomena may not be evident during the initial assessment of the wound, its early detection remains a major unmet challenge. In this article, we demonstrate that digital infrared thermography is a tool that can be used to discriminate burn severity, by detecting changes in temperature at the surface of the skin that is possibly acting as a surrogate for varying degrees of blood supply in the wound. This discrimination capability can be used from the first time the patient is evaluated to guide the clinical decision-making process. We also demonstrate that the prediction capability of the thermograms is very accurate and consistent, suggesting that it can easily be incorporated into clinical practice to establish a more efficient treatment protocol. In this manner, the ΔT parameter has the potential to become a complementary technique for the tactile and visual analysis in the assessment of soft tissue wounds.

**Fig 5:**
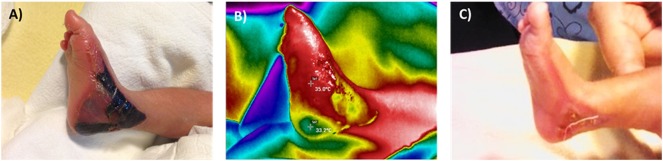
Use of thermography for clinical decision making. Consultation to the burn clinic was requested for a 4-week old infant that had sustained a complete partial thickness burn to his left foot from a heat radiator (A). The paediatric surgeon on call had decided to hospitalize the patient and treat him with a skin graft based on the clinical characteristic of the wound but requested a second opinion to our clinic. The thermographic image showed a ΔT value of 1.8 (B), so conservative management with outpatient management and daily visits to the emergency department to monitor the wound was advised. After seven days of treatment, the wound showed signs of re-epithelization and adequate tissue perfusion. The patient evolution was satisfactory and was discharged from the burn clinic two weeks after the injury.

**Fig 6:**
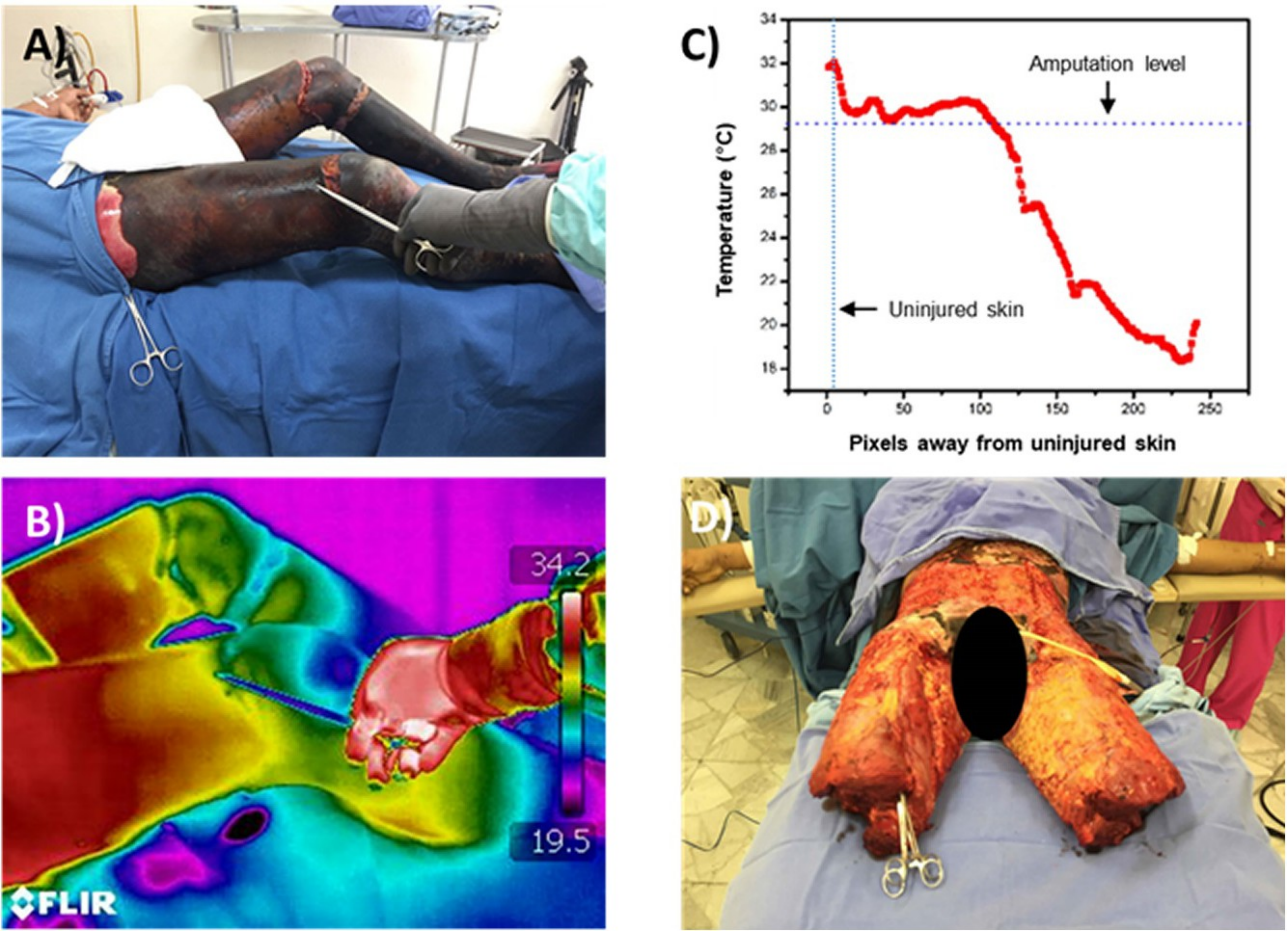
Use of thermography to guide amputation levels. Thermographic imaging can be used as an adjunct to determine amputation levels in severely burned patients. A 24-year old patient with fourth degree burns in approximately 50% of his body surface area because of direct fire was admitted to the burn unit (A). Both legs were severally burned and charred to the clinical inspection. The thermographic image showed progressing ΔT values from 12.7 distally to 1.2 proximally (B, C). A ΔT = 3 was used as a guide to select the amputation level, as it marks the limit for skin grafts and tissue viability. The amputation level is pointed by the forceps on panel A and B. Beyond this level, a sharp decrease in temperature values can be observed in panel C. The patient underwent supracondylar amputation of both legs, as well as tangential excision of all charred skin and was treated with skin grafts. At the moment of publication of this vignette, the patient is still being treated at the burn unit. This approach could also be potentially applied in patients with peripheral vascular disease to promote limb salvage or select optimal levels to create flaps for wound management and future prosthesis fitting.

Thermal imaging technology records the radiation emitted by an object. Heat radiation from skin or underlying injured tissues originates from its blood supply, which if compromised will cause a temperature drop. Previous studies have determined that this imaging method measures the heat emitted from a depth of 1 to 3 mm [12]. Our results and the prediction model we developed are based on the temperature difference between injured and adjacent healthy skin. Thus, the ΔT value offers insight into the extent of tissue injury and the degree of blood supply deficit. This approach has also been used to assess burn depth, finding that as the wound becomes deeper, it also becomes cooler [22]. A more recent study reported that infrared thermal imaging could predict burn depth better than the clinical examination alone through dynamic changes of the wound temperature between days one and two after the injury. As this temperature change over two days likely represents wound conversion, thermography may be an ideal method to detect it. The authors concluded that the overall accuracy of digital infrared thermography for the prediction of wound depth was greater than clinical assessment alone, and that decrease in temperature was predictive of a deeper wound [23]. Moreover, since this temperature drop very likely represents wound conversion, thermography may be an ideal method to detect it. Further advantages of digital thermography is that it is non-invasive, painless, and requires no contact, thus avoiding the risk of contamination and applying pressure to the wound that may affect the microcirculation. Thermograms are rapid to acquire, easy to interpret as they are based on heat maps, and the training required to acquire them is minimal. For these reasons, they could become a useful tool for the early assessment of patients in the emergency department, as well as in the later stages of patient care to identify surface necrosis, distinguish between partial and full-thickness burns and detect complications [6, 24, 25]. Yet, in our opinion, the most relevant contribution of thermography for wound care could be allowing the clinical team to rapidly and objectively determine the treatment modality that is needed, thereby preventing unnecessary procedures or delays in surgery. Currently, the only evidence-based adjunct to clinical evaluation of wound depth and treatment assessment is laser doppler imaging (LDI) [26]. Numerous studies have assessed the utility of LDI, concluding that it reliably discriminates between wounds that will or will not heal by re-epithelization by the third week. The accuracy of this imaging technology compared to the clinical outcome ranges from 90 to 97%, with a positive predictive value as high as 98.4% [2, 3, 27]. While these values are superior to the ones we found, LDI has several major drawbacks that make this technology non-viable for extensive clinical use, such as a high cost for acquiring and maintaining the technology, the need for trained personnel to operate the equipment and interpret the images, and its relatively long time needed to acquire images (i.e. over a minute per scan), therefore requiring sedation of paediatric or non-complying patients to guarantee image quality [28–30]. As thermography circumvents all these drawbacks, we believe its widespread use could be feasible, especially in high-volume centres where there is a compelling need to rationalize patient care, in sites without ready access to a burn surgeon, where thermography may be used to triage patients and help prioritize transfers, or in limited resource settings, where transfers may be difficult to achieve [13, 22, 31]. Two clinical vignettes illustrating the clinical use of the algorithm for helping decide treatment management and amputation levels after significant burns in two independent patients from the study’s cohorts are presented in Figs 5 and 6.

This study has some limitations: first, we only included patients with burns in extremities, so our results cannot be extrapolated to other areas in the body. Refinement of the algorithm to include burn areas in the head or trunk where no amputation is feasible is currently being explored, along with the search for the optimal time of thermography imaging, even though we did not find a significant association between the time of imaging and the ΔT values. Thermal modelling of injured and healthy tissue warrants further exploration, as it could improve our prediction model and offer information on the time to recovery of different types of wounds and in dynamic changes of the wound temperature, thus providing additional data to better rationalize the appropriate treatment for the patient [32]. Second, the algorithm was trained to make the same treatment decisions as the burn surgeons that treated the patients, therefore, even though that the results shown are supported by the k-means clustering algorithm, they may be applicable only to our burn centre. To generalize the results and applicability of the algorithm, further studies in different populations and settings may be required. Third, while it is widely recognized that the depth of burn wounds is not entirely static and that a variety of factors can promote the deepening of a burn in the first few days after sustaining it, not all burns undergo this phenomenon. Ischemia and autophagy of the tissue have been proposed as the leading causes of burn conversion, and as such, it is very likely that infrared thermography can detect it in its early stages. If this is true, wounds with higher ΔT values would probably be associated with burn conversion and deepening of the injury. Nevertheless, in the present study, we did not record burn conversion or dynamic changes in ΔT values, which would have been needed to confirm the hypothesis. It seems that thermography is sensitive enough not to be affected by this factor, but further studies are needed to clarify this point. Finally, a caveat of the method is that we measured ΔT of the wounds as an average, yet most wounds will have areas with different temperature values. Indeed, this approach may give further insights to the clinical team and help streamline the treatment of the patients even more.

Other thermographic techniques for burn assessment are active dynamic infrared thermal imaging (ADT) and Time-Lapse Thermography. These methods are based on infrared detection and show thermal tissue properties instead of changes in temperature distribution. In both techniques, an external thermal stimulus is applied followed by measurements of temperature transients on the tested surface [33–35]. The major drawback of both techniques is that they require additional equipment or interventions to modify the conditions of the burned area. Assessment of ΔT, which relies on static infrared thermographic imaging, have thus several advantages: it doesn't require additional equipment or interventions, its non-invasive and offers the possibility of assessing relatively large body areas. We decided to use static imaging for the sake of simplicity and reproducibility of the method. We believe that by using the least complicated technique, the adoption of our algorithm to the clinical practice and in different settings may be easier to achieve. Perspectives for the use of digital infrared thermography are the assessment of the prediction model in other types of injuries, as a triage tool for emergency rooms or combat situations, and as a telemedicine adjunct in association with a mobile app.

In conclusion, digital infrared thermography can be used as an independent predictor of burn wound healing clinical outcomes such as healing by re-epithelization, requiring a skin graft, or the need of amputation. In this article, we introduce a new prediction algorithm based on the difference of temperature between the injured and healthy tissue that offers the advantages of having a simple and accurate data acquisition protocol in the first days of treatment, and which can easily be incorporated into the current wound management protocols to rationalize treatment.

## Supporting information

S1 Fig Thermogram analysis and ΔT acquisition. Analysis of the thermographic images was done using the FLIR Tools Quick-Report v.1.2 software. The software displays the thermographic image, as well as a clinical phantom (top right corner). A researcher blinded to the clinical characteristics of the wound draw a region of interest (ROI) over the injured area (arrow) and over the adjacent healthy skin (asterisk). The software automatically detects the minimum, maximum (red triangle) and average temperature of both ROI (arrowhead). The difference between the mean temperatures was recorded as the ΔT.
(TIF)

S2 Fig Complexity parameter graph. The complexity parameter (cp) was calculated according to the number of partitioning of the decision tree. A tree with no partitioning (size = 1) has a cp value of infinite, with one partitioning (size = 2) has a cp value of 0.47 and with two partitioning (size = 3) has a value of 0.065. The lower the cp, the lower the relative error of the model to predict the treatment modality. The cp value for the decision tree presented in this paper is 0.065, which corresponds to an X-value relative error of 0.38.
(TIF)

S1 File Dataset. Anonymized data.
(XLSX)

S2 File TRIPOD checklist. (DOCX)

## References

[1] WHO | Burns [Internet]. WHO. [cited 2017 May 18]. Available from: http://www.who.int/mediacentre/factsheets/fs365/en/

[2] Paul DW, Ghassemi P, Ramella-Roman JC, Prindeze NJ, Moffatt LT, Alkhalil A, et al Noninvasive imaging technologies for cutaneous wound assessment: A review. Wound Repair Regen Off Publ Wound Heal Soc Eur Tissue Repair Soc. 2015 4;23(2):149–62. 10.1111/wrr.12262 25832563

[3] Jaskille AD, Shupp JW, Jordan MH, Jeng JC. Critical review of burn depth assessment techniques: Part I. Historical review. J Burn Care Res Off Publ Am Burn Assoc. 2009 12;30(6):937–47. 10.1097/BCR.0b013e3181c07f21 19898102

[4] Jayachandran M, Rodriguez S, Solis E, Lei J, Godavarty A. Critical Review of Noninvasive Optical Technologies for Wound Imaging. Adv Wound Care. 2016 8 1;5(8):349–59. 10.1089/wound.2015.0678 PMC4991615 27602254

[5] Monstrey S, Hoeksema H, Verbelen J, Pirayesh A, Blondeel P. Assessment of burn depth and burn wound healing potential. Burns J Int Soc Burn Inj. 2008 9;34(6):761–9. 10.1016/j.burns.2008.01.009 18511202

[6] Kaiser M, Yafi A, Cinat M, Choi B, Durkin AJ. Noninvasive assessment of burn wound severity using optical technology: a review of current and future modalities. Burns J Int Soc Burn Inj. 2011 5;37(3):377–86. 10.1016/j.burns.2010.11.012 PMC3131405 21185123

[7] Devgan L, Bhat S, Aylward S, Spence RJ. Modalities for the assessment of burn wound depth. J Burns Wounds. 2006 2 15;5:e2 16921415 PMC1687143

[8] Dargaville TR, Farrugia BL, Broadbent JA, Pace S, Upton Z, Voelcker NH. Sensors and imaging for wound healing: a review. Biosens Bioelectron. 2013 3 15;41:30–42. 10.1016/j.bios.2012.09.029 23058663

[9] Miccio J, Parikh S, Marinaro X, Prasad A, McClain S, Singer AJ, et al Forward-looking infrared imaging predicts ultimate burn depth in a porcine vertical injury progression model. Burns J Int Soc Burn Inj. 2016 3;42(2):397–404. 10.1016/j.burns.2015.07.006 26775220

[10] Medina-Preciado JD, Kolosovas-Machuca ES, Velez-Gomez E, Miranda-Altamirano A, González FJ. Noninvasive determination of burn depth in children by digital infrared thermal imaging. J Biomed Opt. 2013 6;18(6):061204 10.1117/1.JBO.18.6.061204 23111601

[11] Martínez-Jiménez MA, Aguilar-García J, Valdés-Rodríguez R, Metlich-Medlich MA, Dietsch LJP, Gaitán-Gaona FI, et al Local use of insulin in wounds of diabetic patients: higher temperature, fibrosis, and angiogenesis. Plast Reconstr Surg. 2013 12;132(6):1015e–9e. 10.1097/PRS.0b013e3182a806f0 24281606

[12] Sagaidachnyi AA, Fomin AV, Usanov DA, Skripal AV. Thermography-based blood flow imaging in human skin of the hands and feet: a spectral filtering approach. Physiol Meas. 2017 2;38(2):272–88. 10.1088/1361-6579/aa4eaf 28099162

[13] Jaspers MEH, Maltha I, Klaessens JHGM, de Vet HCW, Verdaasdonk RM, van Zuijlen PPM. Insights into the use of thermography to assess burn wound healing potential: a reliable and valid technique when compared to laser Doppler imaging. J Biomed Opt. 2016 9 1;21(9):96006 10.1117/1.JBO.21.9.096006 27623232

[14] Liddington MI, Shakespeare PG. Timing of the thermographic assessment of burns. Burns J Int Soc Burn Inj. 1996 2;22(1):26–8. 10.1016/0305-4179(95)00076-3 8719312

[15] Moreira DG, Costello JT, Brito CJ, Adamczyk JG, Ammer K, Bach AJE, et al Thermographic imaging in sports and exercise medicine: A Delphi study and consensus statement on the measurement of human skin temperature. J Therm Biol. 2017 10;69:155–62. 10.1016/j.jtherbio.2017.07.006 29037377

[16] Ammer K. The Glamorgan Protocol for recording and evaluation of thermal images of the human body. Thermol Int. 2008;18:125–44.

[17] Johnson RM, Richard R. Partial-thickness burns: identification and management. Adv Skin Wound Care. 2003 8;16(4):178–87; quiz 188–9. 12897674 10.1097/00129334-200307000-00010

[18] Isbi Practice Guidelines Committee, Steering Subcommittee, Advisory Subcommittee. ISBI Practice Guidelines for Burn Care. Burns. 2016;42(5):953–1021. 10.1016/j.burns.2016.05.013 27542292

[19] Kuhn M. Building Predictive Models in R Using the caret Package. J Stat Softw [Internet]. 2008;28(5). Available from: https://www.jstatsoft.org/article/view/v028i05

[20] Salibian AA, Rosario ATD, Severo LDAM, Nguyen L, Banyard DA, Toranto JD, et al Current concepts on burn wound conversion-A review of recent advances in understanding the secondary progressions of burns. Burns J Int Soc Burn Inj. 2016 8;42(5):1025–35. 10.1016/j.burns.2015.11.007 PMC4947443 26787127

[21] Xiao M, Li L, Li C, Zhang P, Hu Q, Ma L, et al Role of autophagy and apoptosis in wound tissue of deep second-degree burn in rats. Acad Emerg Med Off J Soc Acad Emerg Med. 2014 4;21(4):383–91. 10.1111/acem.12352 PMC4114170 24730400

[22] Hardwicke J, Thomson R, Bamford A, Moiemen N. A pilot evaluation study of high resolution digital thermal imaging in the assessment of burn depth. Burns J Int Soc Burn Inj. 2013 2;39(1):76–81. 10.1016/j.burns.2012.03.014 22652476

[23] Singer AJ, Relan P, Beto L, Jones-Koliski L, Sandoval S, Clark RAF. Infrared Thermal Imaging Has the Potential to Reduce Unnecessary Surgery and Delays to Necessary Surgery in Burn Patients. J Burn Care Res Off Publ Am Burn Assoc. 2016 12;37(6):350–5. 10.1097/BCR.0000000000000330 26720102

[24] Moore K, McCallion R, Searle RJ, Stacey MC, Harding KG. Prediction and monitoring the therapeutic response of chronic dermal wounds. Int Wound J. 2006 6;3(2):89–96. 17007340 10.1111/j.1742-4801.2006.00212.x PMC7951240

[25] Pinzur MS. Outcomes-oriented amputation surgery. Plast Reconstr Surg. 2011 1;127 Suppl 1:241S–247S. 21200297 10.1097/PRS.0b013e318200a409

[26] Jaskille AD, Ramella-Roman JC, Shupp JW, Jordan MH, Jeng JC. Critical review of burn depth assessment techniques: part II. Review of laser doppler technology. J Burn Care Res Off Publ Am Burn Assoc. 2010 2;31(1):151–7. 10.1097/BCR.0b013e3181c7ed60 20061851

[27] Burke-Smith A, Collier J, Jones I. A comparison of non-invasive imaging modalities: Infrared thermography, spectrophotometric intracutaneous analysis and laser Doppler imaging for the assessment of adult burns. Burns J Int Soc Burn Inj. 2015 12;41(8):1695–707. 10.1016/j.burns.2015.06.023 26421694

[28] Hoeksema H, Baker RD, Holland AJA, Perry T, Jeffery SLA, Verbelen J, et al A new, fast LDI for assessment of burns: a multi-centre clinical evaluation. Burns J Int Soc Burn Inj. 2014 11;40(7):1274–82. 10.1016/j.burns.2014.04.024 24996246

[29] Pape SA, Baker RD, Wilson D, Hoeksema H, Jeng JC, Spence RJ, et al Burn wound healing time assessed by laser Doppler imaging (LDI). Part 1: Derivation of a dedicated colour code for image interpretation. Burns J Int Soc Burn Inj. 2012 3;38(2):187–94. 10.1016/j.burns.2010.11.009 22115981

[30] Monstrey SM, Hoeksema H, Baker RD, Jeng J, Spence RS, Wilson D, et al Burn wound healing time assessed by laser Doppler imaging. Part 2: validation of a dedicated colour code for image interpretation. Burns J Int Soc Burn Inj. 2011 3;37(2):249–56. 10.1016/j.burns.2010.08.013 21084164

[31] Chakraborty C, Gupta B, Ghosh SK, Das DK, Chakraborty C. Telemedicine Supported Chronic Wound Tissue Prediction Using Classification Approaches. J Med Syst. 2016 3;40(3):68 10.1007/s10916-015-0424-y 26728394

[32] González FJ. Theoretical and clinical aspects of the use of thermography in non-invasive medical diagnosis. Biomed Spectrosc Imaging. 2016 1 1;5(4):347–58.

[33] Renkielska A, Nowakowski A, Kaczmarek M, Ruminski J. Burn depths evaluation based on active dynamic IR thermal imaging—a preliminary study. Burns J Int Soc Burn Inj. 2006 11;32(7):867–75. 10.1016/j.burns.2006.01.024 16997482

[34] Renkielska A, Kaczmarek M, Nowakowski A, Grudziński J, Czapiewski P, Krajewski A, et al Active dynamic infrared thermal imaging in burn depth evaluation. J Burn Care Res Off Publ Am Burn Assoc. 2014 10;35(5):e294–303. 10.1097/BCR.0000000000000059 25144810

[35] Simmons JD, Kahn SA, Vickers AL, Crockett ES, Whitehead JD, Krecker AK, et al Early Assessment of Burn Depth with Far Infrared Time-Lapse Thermography. J Am Coll Surg. 2018 4;226(4):687–93. 10.1016/j.jamcollsurg.2017.12.051 29409904 PMC5880281

