# Supplementary figures and images for: Development and validation of an algorithm to predict the treatment modality of burn wounds using thermographic scans: Prospective cohort study
Source: PLoS One. 2018 Nov 14;13(11):e0206477. doi: 10.1371/journal.pone.0206477 (PMC6235294; doi:10.1371/journal.pone.0206477)

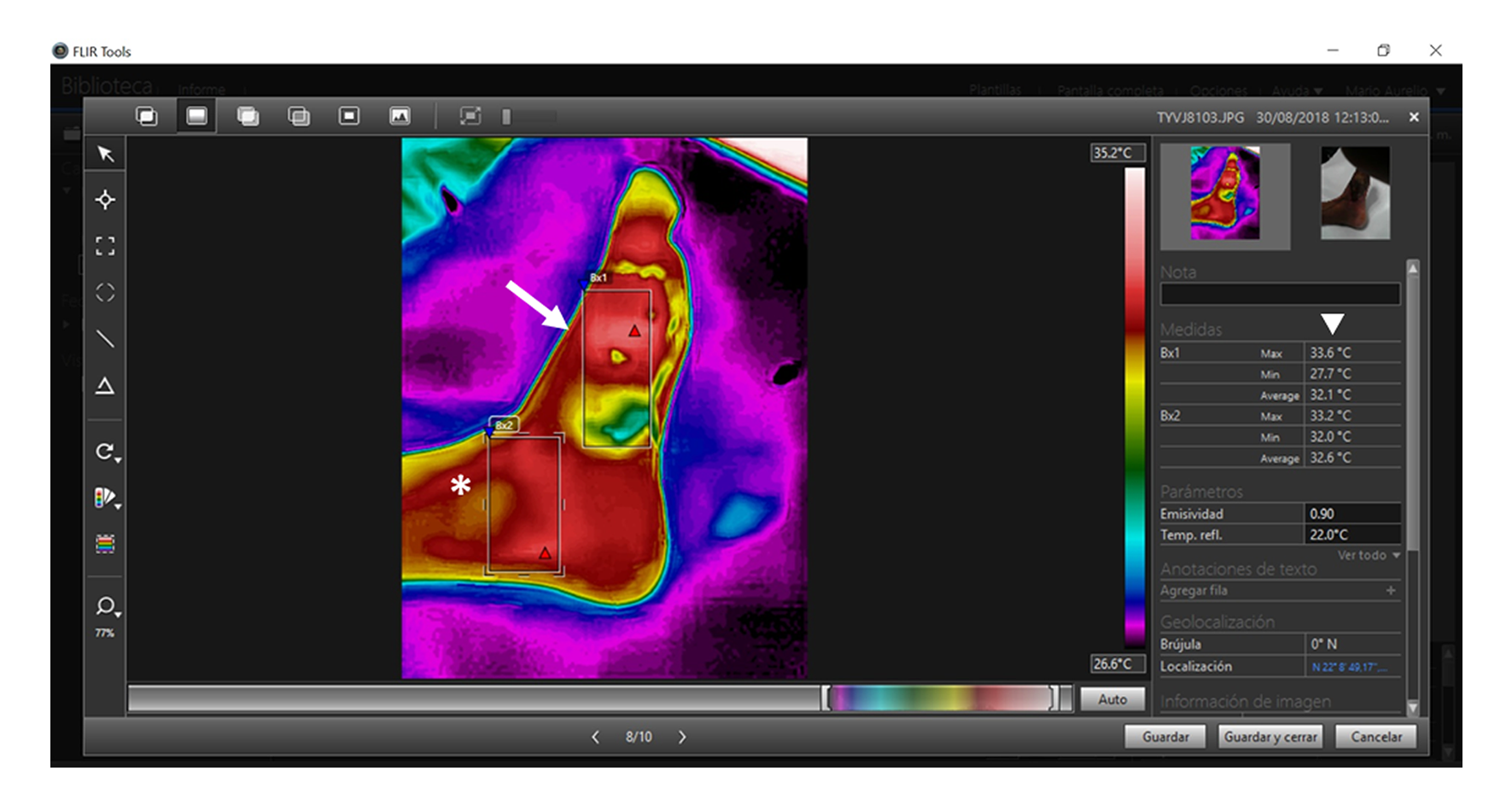

Supplement: S1 Fig — Analysis of the thermographic images was done using the FLIR Tools Quick-Report v.1.2 software. The software displays the thermographic image, as well as a clinical phantom (top right corner). A researcher blinded to the clinical characteristics of the wound draw a region of interest (ROI) over the injured area (arrow) and over the adjacent healthy skin (asterisk). The software automatically detects the minimum, maximum (red triangle) and average temperature of both ROI (arrowhead). The difference between the mean temperatures was recorded as the ΔT. (TIF) [file pone.0206477.s001.tif]

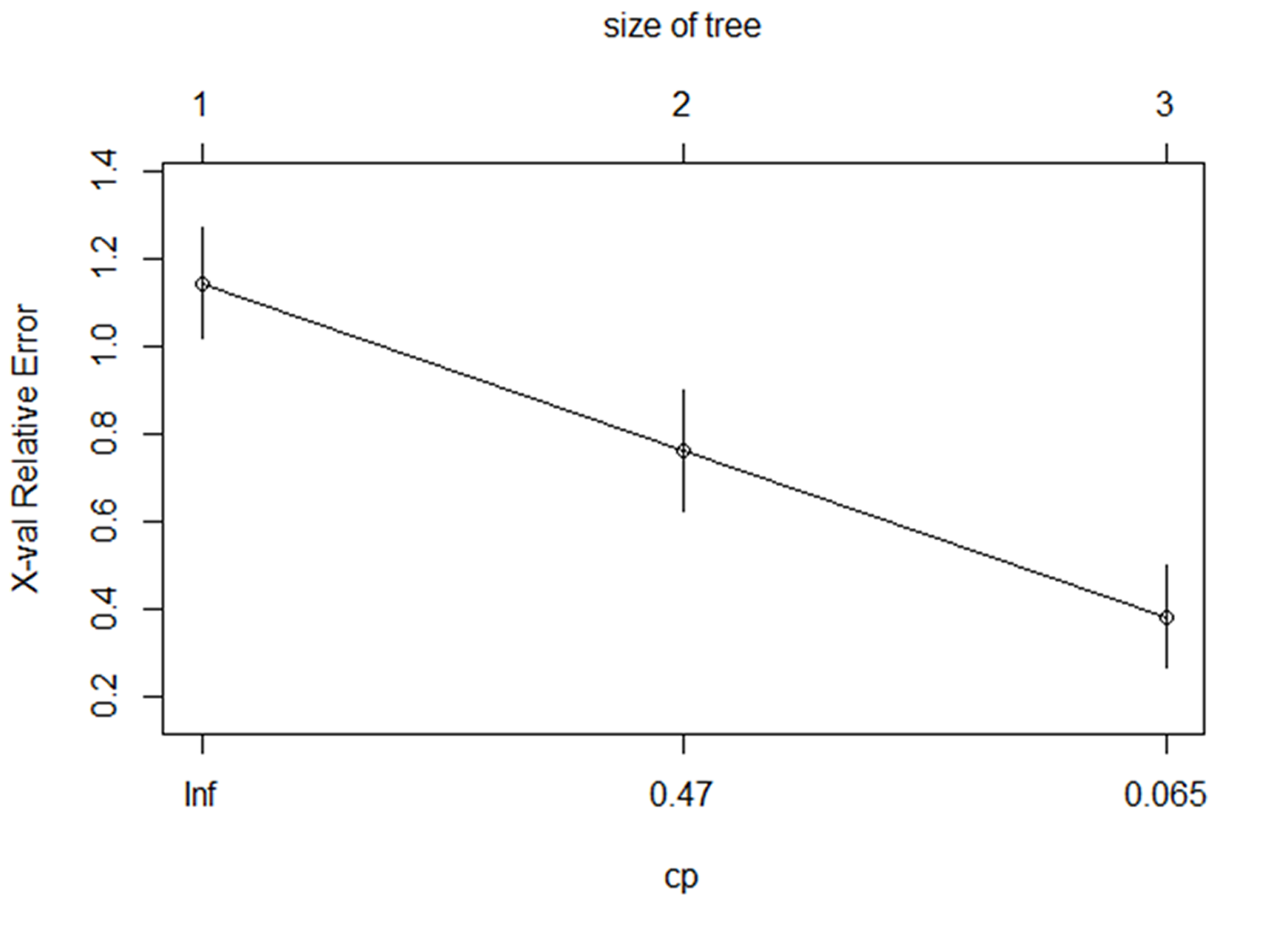

Supplement: S2 Fig — The complexity parameter (cp) was calculated according to the number of partitioning of the decision tree. A tree with no partitioning (size = 1) has a cp value of infinite, with one partitioning (size = 2) has a cp value of 0.47 and with two partitioning (size = 3) has a value of 0.065. The lower the cp, the lower the relative error of the model to predict the treatment modality. The cp value for the decision tree presented in this paper is 0.065, which corresponds to an X-value relative error of 0.38. (TIF) [file pone.0206477.s002.tif]
