# Supplementary material for: Development and validation of an algorithm to predict the treatment modality of burn wounds using thermographic scans: Prospective cohort study
Source: PLoS One. 2018 Nov 14;13(11):e0206477. doi: 10.1371/journal.pone.0206477 (PMC6235294; doi:10.1371/journal.pone.0206477)
Supplement: S2 File — (DOCX) [file pone.0206477.s004.docx]

| **Section/Topic** | **Item** |  | **Checklist Item** | **Page** |
| --- | --- | --- | --- | --- |
| **Title and abstract** | | | | |
| Title | 1 | D;V | Identify the study as developing and/or validating a multivariable prediction model, the target population, and the outcome to be predicted.  **Development and validation of an algorithm to predict the treatment modality of burn wounds using thermographic scans: prospective cohort study** | 1 |
| Abstract | 2 | D;V | Provide a summary of objectives, study design, setting, participants, sample size, predictors, outcome, statistical analysis, results, and conclusions. | 3 |
| **Introduction** | | | | |
| Background and objectives | 3a | D;V | Explain the medical context (including whether diagnostic or prognostic) and rationale for developing or validating the multivariable prediction model, including references to existing models.  **Digital infrared thermography imaging can be used to assess the severity of burns in a non-invasive manner. This imaging modality may provide more information about the degree of tissue damage than clinical inspection alone during the various phases of wound healing and can quantitatively assess the burn depth based on the digital images acquired.** | 4-5 |
|  | 3b | D;V | Specify the objectives, including whether the study describes the development or validation of the model or both.  **In this study, we developed a model to predict the treatment modality needed to promote closure of extensive skin burns in limbs based on thermographic imaging of the wound obtained during the first three days of treatment. Our hypothesis was that the temperature difference between the healthy and wounded skin correlates with the required definitive treatment in a cohort of burn patients. We categorized the treatment as “re-epithelization” if the wound healed spontaneously, “skin graft” if the injury healed after receiving a graft, or “amputation” if the patient required removal of part of an appendage because of lack of tissue viability. Subsequently, by using the ΔT values, we created a prediction model based on temperature difference cut offs for each treatment modality. Finally, we tested and validated the prediction model in an independent cohort of similarly burned patients.** | 5 |
| **Methods** | | | | |
| Source of data | 4a | D;V | Describe the study design or source of data (e.g., randomized trial, cohort, or registry data), separately for the development and validation data sets, if applicable.  **This was a prospective observational study.**  **We prospectively enrolled 22 new patients with similar characteristics to develop a validation cohort. Clinical characteristics and their comparison to the development cohort are shown on Table 1.** | 5, 18 |
|  | 4b | D;V | Specify the key study dates, including start of accrual; end of accrual; and, if applicable, end of follow-up.  **Infrared thermography was performed once within the first 3 days after injury.**  **The flowchart summary of the study is shown in figure 1.** | 6, 9, 11 |
| Participants | 5a | D;V | Specify key elements of the study setting (e.g., primary care, secondary care, general population) including number and location of centres.  **All study patients received treatment at the burn care unit of Hospital Central “Dr. Ignacio Morones Prieto”, a major referral centre for burn injuries in central Mexico.** | 6 |
|  | 5b | D;V | Describe eligibility criteria for participants.  **Inclusion criteria were patients having sustained a partial or full thickness burns in extremities covering >25 cm2 of total body surface and who were admitted to the burn care unit within 24 hours from injury. Exclusion criteria were presence of any previous comorbidity, a baseline body mass index of <19.9 for adults or below the 5th percentile for their age in children, presence of foreign bodies embedded in the tissue, gross oedema, systemic causes of distal hypoperfusion, and/or presence of local infection. No patients were eliminated from the study after enrolment.** | 6 |
|  | 5c | D;V | Give details of treatments received, if relevant.  **All burns received the standard treatment according to the International Society for Burn Injuries guidelines by a surgical team blinded to the thermograms and/or prediction data: cleansing the wound every 72 hours, early excision of necrotic tissue, wound coverage with silver sulfadiazine, and no antibiotic prophylaxis** | 7 |
| Outcome | 6a | D;V | Clearly define the outcome that is predicted by the prediction model, including how and when assessed.  **The modality of wound treatment was defined as “re-epithelization” if the wound re-epithelized by itself before 15 days of care; “skin graft” if the wound healed after receiving one or more skin grafts (all patients received auto-grafts), or “amputation”, if the appendage was removed. We recorded the final wound modality, for example if a wound received a graft but the extremity became nonviable and was amputated, it was considered as an amputation.** | 7-8 |
|  | 6b | D;V | Report any actions to blind assessment of the outcome to be predicted.  **After admission to the burn care unit and enrolment into the study, thermograms were acquired and analysed by an independent member of the research team. The surgical team was kept blinded to the prediction results until discharge of the patients, when their treatment modality was recorded.** | 18 |
| Predictors | 7a | D;V | Clearly define all predictors used in developing or validating the multivariable prediction model, including how and when they were measured.  **The model had three classes (re-epithelization, skin graft or amputation) and 5 predictors (ΔT, age, burn aetiology, depth of injury, and burn area).** | 14-17 |
|  | 7b | D;V | Report any actions to blind assessment of predictors for the outcome and other predictors.  **To create the decision-making model, we used the Classification and regression training (caret) package for R to create and validate a predictive model to assign a treatment to patients based on their ΔT.** | 16 |
| Sample size | 8 | D;V | Explain how the study size was arrived at.  **A power analysis was performed based on results of a previous study [12]. We determined that a minimum of 10 patients per outcome group were needed in order to detect a difference of 2.0 ± 1.5 °C between treatment groups at an alpha level of 95% and a statistical power of 80%.** | 8 |
| Missing data | 9 | D;V | Describe how missing data were handled (e.g., complete-case analysis, single imputation, multiple imputation) with details of any imputation method. | N/A |
| Statistical analysis methods | 10a | D | Describe how predictors were handled in the analyses.  **To create the decision-making model, we used the Classification and regression training (caret) package for R to create and validate a predictive model to assign a treatment to patients based on their ΔT.** | 16 |
|  | 10b | D | Specify type of model, all model-building procedures (including any predictor selection), and method for internal validation.  **Potential confounding factors associated to ΔT measurements were assessed through bi-variate linear analysis.**  **To create the decision-making model, we used the Classification and regression training (caret) package for R to create and validate a predictive model to assign a treatment to patients based on their ΔT.**  **To test the prediction accuracy of the model in the development cohort, we obtained the inter-rater agreement weighted kappa coefficient.** | 14-17 |
|  | 10c | V | For validation, describe how the predictions were calculated.  **To test the prediction accuracy of the model in the development cohort, we obtained the inter-rater agreement weighted kappa coefficient. The algorithm predicted that 14 patients would heal by re-epithelization, 9 through skin grafts, and 11 would require an amputation. The treatment modality used on the patients was conservative treatment and re-epithelization in 13, skin graft in 10, and amputation in 11, thus the algorithm misclassified 3 patients (one in the re-epithelization group and two on the skin graft group). Agreement rate between the prediction and the patient outcome was weighted kappa = 0.904 (p <0.001).**  **After admission to the burn care unit and enrolment into the study, thermograms [of the validation cohort] were acquired and analysed by an independent member of the research team. The surgical team was kept blinded to the prediction results until discharge of the patients, when their treatment modality was recorded. The algorithm predicted that 9 patients would heal with by re-epithelization with conservative treatment, 6 through skin grafts, and 7 would require an amputation. The outcome of the patients was re-epithelization in 9, skin graft in 6, and amputation in 7; thus, the algorithm misclassified 2 patients (Table 4). Agreement rate between the prediction and the patient outcome was weighted kappa = 0.901 (p <0.001).** | 17-18 |
|  | 10d | D;V | Specify all measures used to assess model performance and, if relevant, to compare multiple models.  **For the development of the prediction model, receiver-operator characteristic (ROC) curves were used, as well as machine learning classification and regression training algorithms. Finally, to test the agreement rate between the prediction model and the treatment modality we used weighted kappa analysis.** | 8 |
|  | 10e | V | Describe any model updating (e.g., recalibration) arising from the validation, if done. | N/A |
| Risk groups | 11 | D;V | Provide details on how risk groups were created, if done. | N/A |
| Development vs. validation | 12 | V | For validation, identify any differences from the development data in setting, eligibility criteria, outcome, and predictors.  **We prospectively enrolled 22 new patients with similar characteristics to develop a validation cohort. Clinical characteristics and their comparison to the development cohort are shown on Table 1.** | 18 |
| **Results** | | | | |
| Participants | 13a | D;V | Describe the flow of participants through the study, including the number of participants with and without the outcome and, if applicable, a summary of the follow-up time. A diagram may be helpful.  **Figure 1** | 11 |
|  | 13b | D;V | Describe the characteristics of the participants (basic demographics, clinical features, available predictors), including the number of participants with missing data for predictors and outcome.  **Table 1** | 9-10 |
|  | 13c | V | For validation, show a comparison with the development data of the distribution of important variables (demographics, predictors and outcome).  **Table 1, Table 3** | 9-10, 19 |
| Model development | 14a | D | Specify the number of participants and outcome events in each analysis.  **A total of 34 patients (mean age 26.5 ± 19.4 years, min 1, max 68) were used to develop the prediction model.**  **Patients were followed-up until discharge and their outcome registered. Thirteen patients (37%) healed by re-epithelization, 10 (30%) received skin grafts, and 11 (33%) required an amputation.** | 12 |
|  | 14b | D | If done, report the unadjusted association between each candidate predictor and outcome. | N/A |
| Model specification | 15a | D | Present the full prediction model to allow predictions for individuals (i.e., all regression coefficients, and model intercept or baseline survival at a given time point).  **Figure 3** | 17 |
|  | 15b | D | Explain how to the use the prediction model.  **Figure 3, Figure 4, Figure 5** | 17, 22-23 |
| Model performance | 16 | D;V | Report performance measures (with CIs) for the prediction model.  **We tested the diagnostic accuracy of the model with 100 bootstrap resamples and found an accuracy of 85.35% (95%CI 72.2 to 98.5%) for diagnostic classification. The algorithm misclassified 13.0% of the patients to conservative treatment, 13.0% to skin graft, and 0% to amputation.**  **[On the development cohort] agreement rate between the prediction and the patient outcome was weighted kappa = 0.904 (p <0.001).**  **[On the validation cohort] agreement rate between the prediction and the patient outcome was weighted kappa = 0.901 (p <0.001).** | 16-18 |
| Model-updating | 17 | V | If done, report the results from any model updating (i.e., model specification, model performance). | N/A |
| **Discussion** | | | | |
| Limitations | 18 | D;V | Discuss any limitations of the study (such as nonrepresentative sample, few events per predictor, missing data).  **A potential limitation to this study is that we only included patients with burns in extremities, so our results cannot be extrapolated to other areas in the body.** | 24 |
| Interpretation | 19a | V | For validation, discuss the results with reference to performance in the development data, and any other validation data.  **We demonstrate that the prediction capability of the thermograms is very accurate and consistent** | 20 |
|  | 19b | D;V | Give an overall interpretation of the results, considering objectives, limitations, results from similar studies, and other relevant evidence. | 19-24 |
| Implications | 20 | D;V | Discuss the potential clinical use of the model and implications for future research. | 19-24 |
| **Other information** | | | | |
| Supplementary information | 21 | D;V | Provide information about the availability of supplementary resources, such as study protocol, Web calculator, and data sets. | N/A |
| Funding | 22 | D;V | Give the source of funding and the role of the funders for the present study.  **No specific funding was used for this research project. JLRGL receives doctoral support from the Mexican National Council for Science and Technology (CONACYT) and the Fonds de Recherche en Santé Québec (FRSQ). The funding organizations played no role in the study design; in the collection, analysis, or interpretation of data; in the writing of the report; or in the decision to submit the report for publication.** | 2 |

*Items relevant only to the development of a prediction model are denoted by D, items relating solely to a validation of a prediction model are denoted by V, and items relating to both are denoted D;V. We recommend using the TRIPOD Checklist in conjunction with the TRIPOD Explanation and Elaboration document.
